# Role of mechanoregulation in mast cell-mediated immune inflammation of the smooth muscle in the pathophysiology of esophageal motility disorders

**DOI:** 10.1152/ajpgi.00258.2023

**Published:** 2024-01-30

**Authors:** Raj K. Goyal, Satish Rattan

**Affiliations:** ^1^Division of Gastroenterology, Department of Medicine, Veterans Affairs Boston Healthcare System, West Roxbury, Massachusetts, United States; ^2^Division of Gastroenterology, Hepatology, and Endoscopy, Department of Medicine, Beth Israel Deaconess Medical Center, and Harvard Medical School, Boston, Massachusetts, United States; ^3^Department of Medicine, Division of Gastroenterology and Hepatology, Sidney Kummel Medical College of Thomas Jefferson University, Philadelphia, Pennsylvania, United States

**Keywords:** immune inflammation-mediated changes in smooth muscles, mast cells/smooth muscle cells cytokines, mechanotranslation of smooth muscle hypertrophy and hypercontractility, obstructed bolus transport led mechanical stress and immune inflammation, pathogenesis of esophageal motility disorders

## Abstract

Major esophageal disorders involve obstructive transport of bolus to the stomach, causing symptoms of dysphagia and impaired clearing of the refluxed gastric contents. These may occur due to mechanical constriction of the esophageal lumen or loss of relaxation associated with deglutitive inhibition, as in achalasia-like disorders. Recently, immune inflammation has been identified as an important cause of esophageal strictures and the loss of inhibitory neurotransmission. These disorders are also associated with smooth muscle hypertrophy and hypercontractility, whose cause is unknown. This review investigated immune inflammation in the causation of smooth muscle changes in obstructive esophageal bolus transport. Findings suggest that smooth muscle hypertrophy occurs above the obstruction and is due to mechanical stress on the smooth muscles. The mechanostressed smooth muscles release cytokines and other molecules that may recruit and microlocalize mast cells to smooth muscle bundles, so that their products may have a close bidirectional effect on each other. Acting in a paracrine fashion, the inflammatory cytokines induce genetic and epigenetic changes in the smooth muscles, leading to smooth muscle hypercontractility, hypertrophy, and impaired relaxation. These changes may worsen difficulty in the esophageal transport. Immune processes differ in the first phase of obstructive bolus transport, and the second phase of muscle hypertrophy and hypercontractility. Moreover, changes in the type of mechanical stress may change immune response and effect on smooth muscles. Understanding immune signaling in causes of obstructive bolus transport, type of mechanical stress, and associated smooth muscle changes may help pathophysiology-based prevention and targeted treatment of esophageal motility disorders.

## Graphical abstract

**Figure:**
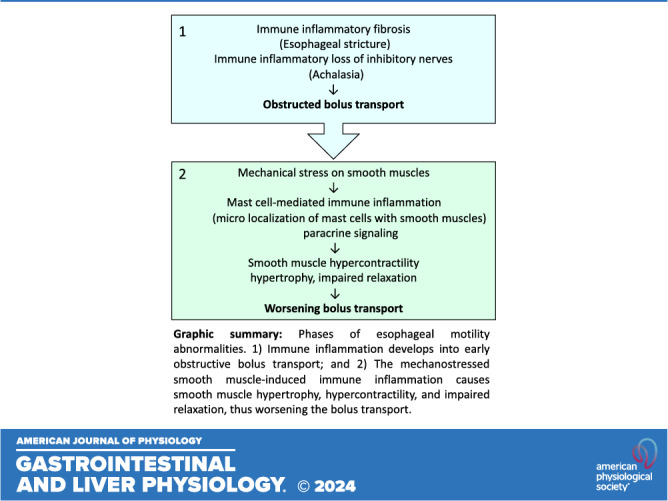

## INTRODUCTION

The human esophagus is composed of two distinct regions: the upper or the cervical esophagus, primarily made up of striated muscles, and the lower or the thoracic esophagus, composed of smooth muscles. Consequently, the neuromuscular organization and the mechanism of peristaltic contractions in the two parts are very different. Still, the swallow-induced “primary” peristaltic contraction travels seamlessly through both the parts to transport food bolus to the stomach (1). Additionally, the esophagus propels material refluxed from the stomach back into the stomach using “secondary” peristalsis.

Subjects with esophageal disorders have symptoms of dysphagia, chest pain, gastroesophageal reflux disease (GERD), and complications such as esophageal adenocarcinoma and pulmonary disease (2, 3). Broadly speaking, the symptoms of dysphagia include all difficulties associated with swallowing. Dysphagia due to obstruction to the passage of food bolus may be due to mechanical constriction of the esophagus or the loss of deglutitive inhibition. Obstructed transport in the esophagus, as in other parts of gut, is associated with smooth muscle contractility and structural changes. These changes may contribute to poor esophageal transport, dysphagia, and chest pain. The pathogenesis of muscle changes is poorly understood.

There is now growing evidence that mechanical and motor causes of labored bolus transport may involve several factors, including immune inflammation. Immune inflammation has been recognized as an important factor in the pathogenesis of intestinal motility disorders for some time (4, 5) but only recently in the esophageal stricture and achalasia (6, 7).

Bolus transport in partially obstructed esophagus due to stricture or achalasia may cause smooth muscle stress and activate the immune system, leading to muscle hypercontractility and hypertrophy. The immune system is usually associated with a response to external infections and allergies (8). Recently, endogenous oxidative stress has been shown to activate macrophage-mediated immune inflammation, resulting in gastric emptying disorders in diabetes (9–11).

Mechanostressed smooth muscles may activate immune inflammation (12). Intestinal smooth muscle releases numerous cytokines and other molecules that can activate mast cells (MCs), which leads to immune inflammation (13, 14). The MC activation may occur via a distinct nonallergic microlocalization and paracrine signaling, which is different from the allergic or inflammatory signaling, responsible for the initial obstruction to bolus transport (15). Macrophages and MCs are now considered key players in the maintenance of gastrointestinal smooth muscle homeostasis, and in disease (16).

The purpose of this review is to explore the involvement of mechanotransduction-associated immune inflammation in the causation of smooth muscle changes in obstructive esophageal bolus transport.

## MECHANOTRANSDUCTION, MECHANOREGULATION, AND MECHANICAL STRESS

Mechanotransduction is the process of transforming changes in force into signals that regulate cellular structure and function (17, 18). Smooth muscle cells (SMCs) perform two primary functions, the contractile and secretory. Myogenic reflex is a rapid contraction that occurs following a stretch of the isolated nerve-free smooth muscle. Mechanical stimuli act on smooth muscles through mechanoreceptors such as integrin, focal adhesion, and a complex cytoskeleton component, including the nuclear cytoskeleton (19). Mechano-gated ion channels, such as the Piezo nonselective cation channels, are responsible for their physiological actions. These ion channels regulate the entry of Ca^2+^ and other ions into the cell and within the cellular compartments. Consequently, they play a crucial role in the regulation of chemical signaling. Interestingly, a variety of ion channels are mechanosensitive but are not mechano-gated (18).

Recently, considerable progress has been made in comprehending the mechanisms underlying mechano-sensing and mechanotransduction (12). Individual or groups of panels of ion channels are involved in mechano-sensing in different reflexes in diverse tissues. Filamin A, an actin-binding protein, also plays a significant role in the regulation of mechano-gated channels (20). In addition, mechanotransduction regulates the secretory function of SMCs, determining the development of either noncontractile (secretory) or contractile phenotypes. Mechano-gated nuclear membrane pores also regulate the trafficking of transcription factors to the nucleus. Furthermore, mechanical stress induces SMCs to secrete cytokines and other alarmins as the initiators of immune inflammation.

## EFFECT OF MECHANICAL STRESS ON ESOPHAGEAL SMOOTH MUSCLE

In a series of studies, Gabella (21) reported that mechanical stress causes muscle hypertrophy and major morphological changes in SMCs. In vivo studies in the opossum esophagus by Mittal et al. (22) reported that an acute partial obstruction causes significant changes in esophageal motility. Tung and colleagues (23) found that the chronic mechanical stress associated with partial obstruction also leads to difficult esophageal transport and esophageal stasis. In these studies, esophageal obstruction was created by placing a loose Gore-Tex band around the esophagogastric junction. These studies revealed an enlargement in the luminal diameter and thickening of the circular muscle in the dilated esophagus, above the obstruction (24). The SMCs exhibited a substantial enlargement, with a cell surface area increase of approximately 60%, without any apparent hyperplasia. The SMCs underwent pleomorphism and envelopment by an amorphous ground substance in the extracellular matrix, devoid of fibrotic changes.

Under electron microscopy, MCs and basophils were observed making close contact with muscle bundles and discharging their granular contents in either the cytoplasm or the extracellular space (25). These observations show that mechanical stress in vivo activates the immune system, responsible for hypercontractility and hypertrophy in the smooth muscles. Mechanical stress may also induce visceral hypersensitivity (26), damage to the inhibitory nerves (27), and loss of neutrally-mediated smooth muscle hyperpolarization (28).

**Figure 1.**
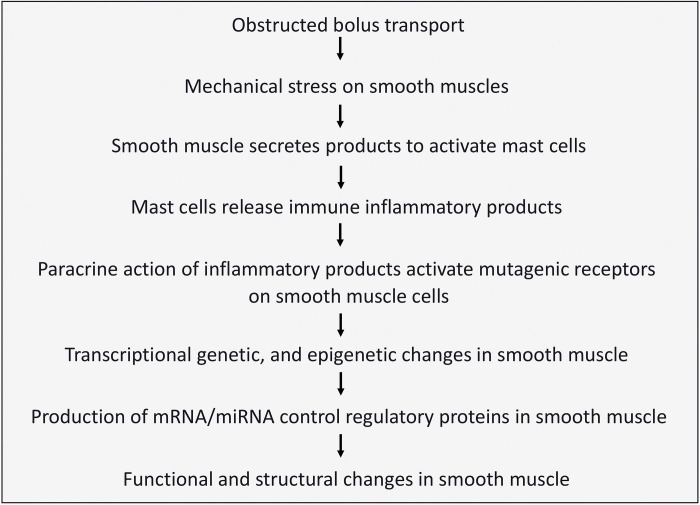
Stepwise pathogenesis of esophageal smooth muscle disorders. Generation of mechanical stress in the esophageal smooth muscles, and stepwise progression of the mechanostressed smooth muscles, leading to functional and structural changes.

Despite major advances in the role of immune inflammation in the regulation of the structure and function of the smooth muscles in other systems, there is a paucity of work on the gastrointestinal smooth muscles, especially in the esophagus. The relevant findings from other systems suggest the possible signaling pathway for the mechanical stress leading to the pathogenesis of esophageal motility disorders (29, 30). Figure 1 lists the steps in the generation of mechanostress and in the progression of mechanostressed smooth muscle-induced functional and structural changes.

## ROLE OF MECHANICAL STRESS-INDUCED IMMUNE INFLAMMATION ON SMOOTH MUSCLE: LESSONS FROM STUDIES ON AIRWAY SMOOTH MUSCLES

In asthma, two distinct immune inflammatory processes coexist:

The first process is mucosal and submucosal infiltrations with eosinophils and MCs, with the expression of interleukin-4 (IL-4), interleukin-5 (IL-5), and T cells. MCs play a crucial role in the release of mediators during the acute phase, marked by significant elevations in histamine, PGD2, tryptase, and occasionally leukotriene C4 (LTC4). Eosinophils are responsible for the secretions of many mediators, such as histamine, LTC4, and eosinophil-derived proteins, during the late phase; Basophils, however, have also been detected at specific time points using morphological and phenotypic criteria (31). As the disease worsens, neutrophils may replace eosinophils. The initiation of a neutrophilic response activates thymic stromal lymphoprotein (TSLP) and TH17 cells. Mucosal and submucosal eosinophilic infiltration also occurs in eosinophilic bronchitis. However, although asthma is characterized by significant airflow difficulty, subjects with eosinophilic bronchitis had little airflow difficulty (32, 33). These studies suggested that mucosal inflammation is not a major cause of airflow difficulty in asthma (32, 33).

The second is in contrast to the combined infiltration of MCs and eosinophils in the mucosa and submucosa, MCs alone (without eosinophils) infiltrate the ASM bundles in asthma. These observations suggested that MC-mediated inflammation of the airway smooth muscle (ASM) may be the primary cause of significant airflow obstruction (32, 33). The mucosal inflammation may cause mechanical stress on the smooth muscles, initiating MC-mediated immune inflammation, which then leads to enhanced ASM reactivity and muscle hypertrophy, worsening the airflow difficulty.

## MAST CELLS

MCs possess numerous receptors that play a crucial role in their recruitment to tissues, localization, and adherence to the target cells. A variety of ion channels are known to regulate intracellular Ca^2+^ ([Ca^2+^]_i_) profile. In addition, the MCs contain a diverse array of presynthesized and stored products with the ability to synthesize new molecules as required. The presynthesized substances comprise vasoactive amines like histamine, proteases like tryptase and chymase, specific cytokines like tumor necrosis factorα (TNFα), and growth factors such as vascular endothelial growth factor (VEGF). The de novo synthesis pathway produces several compounds, such as prostaglandins (PGD2, PGE2), leukotrienes (LTD4, LTE4), interleukins (IL-4, IL-5, IL-6, IL-8), and granulocyte-macrophage colony-stimulating factor (GM-CSF).

The secretory products of MCs exhibit variability among distinct populations of MCs that exert their effects on the ASM. These products include tryptase, transforming growth factor β1 (TGFβ1), basic fibroblast growth factor (βFGF), histamine, interleukin IL-4, and IL-13, prostaglandin D2 (PGD2), and LTC4. The release of these products is achieved through complete degranulation, wherein selective granules are liberated through a process of piecemeal degranulation (34). In this process, selected products via a specific mechanism, the secretion-altered, granules are retained in the cell (35). These processes may involve complete or kiss-and-run type exocytosis (36). Furthermore, various activation stimuli elicit geographically and temporally diverse patterns of granule secretion.

Distinct phenotypes of MCs with selective receptors, signaling, and secretions are involved in the specific inflammatory processes at different stages of the same disease (15).

## MAST CELL PHENOTYPES: ACTIVATION AND SIGNALING

MCs are found in two different locations in asthma: the mucosa and the smooth muscle. In allergic asthma, the mucosal cell secretion of a well-defined panel of chemoattractants and stimulants [e.g., interleukins, IL-33 and IL-1α, ATP, heat shock proteins, HMGB1 (high mobility group box1), and S100 A/B proteins] recruit and activate MCs rich in FcɛR1 (a tetrameric receptor complex that binds the Fc portion of the ε heavy chain of IgE), which are positive mucosal MCs accompanying eosinophilia. This pathway is stimulated by cross linking of FcɛR1 receptors on the MCs by substantial amounts of immunoglobulin E (IgE) antibodies produced by B lymphocytes/plasma cells. This pathway releases numerous pro-inflammatory mediators in enormous quantities, such as PGE2 (prostaglandin E2), cytokines, and VEGF. This secretory profile is associated with prolonged Ca^2+^ release, granule fusion into large irregularly shaped granules, and exocytosis via SNAP23-STX-4 complexes (37).

The MRGPRX2 receptor, also known as Mas-related G protein-coupled receptor (GPCR) family member 2, is a newly discovered receptor on the MCs (38). The stimulation of this receptor is initiated by several endogenous peptides, bacterial products, and synthetic medicines. This results in the selective, rapid and transient release of small vesicle-associated mediators (histamine, heparin proteases), prostaglandins, leukotrienes, cytokines, and chemokines. This pathway participates in the IgE-independent pseudo-allergic- and drug hypersensitivity reactions (35, 38, 39). Several other pathways of MC signaling have been identified that translate sensing of changes into specific responses (40, 41).

**Figure 2.**
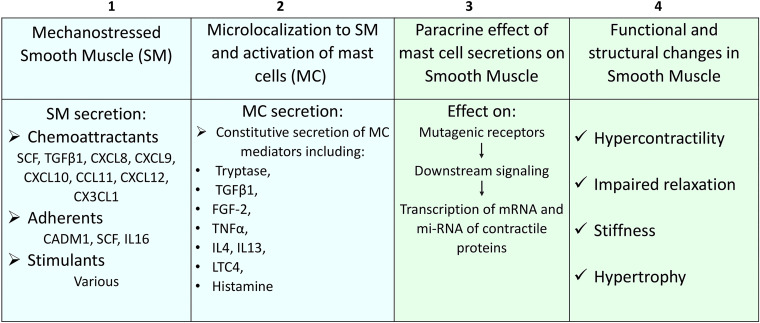
Mechanotranslation to hypertrophy, and hypercontractility in the esophageal smooth muscle. The mechanostressed smooth muscles above the obstruction, secrete chemoattractants, adherents like CADM1, and various stimulants lead to recruit and microlocalize mast cells to smooth muscle bundles (*boxes 1* and *2*). This is followed by their stimulation to secrete mediators, which act in a paracrine fashion on the smooth muscle to transcribe contractile proteins, leading (*box 3*) to functional and structural changes such as hypercontractility, impaired relaxation, stiffness, and hypertrophy (*box 4*). However, under different types of mechanoobstruction, mast cells may secrete inhibitory mediators causing muscle hypocontractility and atrophy.

The smooth muscle MCs are located deep in the tissues and, unlike epithelial cells, are not exposed to external allergens. Figure 2 summarizes the signaling pathway of mechanostress to functional and structural changes in the smooth muscle. Mechanostressed smooth muscles may release a panel of chemoattractant molecules, such as SCF, TGF1, CXCL8-9-10, 12, CX3CL1, and chemokine (C-C motif) ligand 11 (CCL11), which bind to the MCs’ respective receptors to recruit them. Subsequently, microlocalization is achieved through cell adhesive molecule1 (CADM1), which adheres the mast cell with the smooth muscle (42, 43). This promotes adherence through the interaction between membrane-bound, colony-stimulating factor (CSF) on SMCs and its receptor c-kit on MCs, as well as IL-6. Because of the specificity of the chemoattractants and the adhesive molecules secreted by the SMCs, only a selective population of MCs may be recruited.

The MCs are a heterogeneous population of highly adaptable cells that adjust their shape according to their environment (44). Microlocalization brings MCs closer to the stressed smooth muscles within 2 nm of each other, a closer proximity than that of ∼4 nm in a neural synapse (29, 30, 34, 36). The process of microlocalization facilitates the intimate bidirectional communication between the products of SMCs and those of the MCs (45). The cooperative function of MCs contributes to cell survival, proliferation, and constitutive activation (46). The microlocalized MCs have their specific receptors and the secretory profiles appropriate for their function. The mechanostressed smooth muscles release interleukins such as IL-33, IL-1α, and extracellular ATP, activating MCs via their respective receptors (47). Only a few MCs contact a muscle bundle of several dozen SMCs that are interconnected by gap junctions. In addition, the symptomatic responsiveness of asthma to corticosteroids, as well as β2-adrenoceptor agonist (48, 49), dependent on the state of immune inflammation, suggests the role of immune inflammation in the ASM.

## REGULATION OF TRANSCRIPTOME, AND EPIGENOME OF SMOOTH MUSCLE

The secretory products of MCs activate one or more GPCR, receptor tyrosine kinase (RTK), or the cytokine receptors coupled with cytosolic nonreceptor tyrosine kinase Janus Kinase (JAK) (34). Stimulation of these receptors and production of transcription factors can turn genes “on” and “off.” Cytokine receptors signal via JAK/STAT (signal transducers and activators of transcription). Therefore, JAK/STAT signaling holds significant therapeutic potential (50). The downstream signaling pathway of RTK may involve multiple pathways.

The process of transcribing entails the interactions between a transcription factor and a part of the DNA, affecting a change in the mRNA and hence in the remodeling of proteins. In this regard, transcription factors play a crucial role in the regulation of the epigenome and the production of micro-RNAs (mi-RNAs), small (∼22 nucleotides) noncoding RNA molecules, transcribed by RNA polymerases, which generate precursors for the mature mi-RNAs (51). The latter interacts with mRNA to primarily downregulate the expression of the corresponding protein. In some cases, the effects of mi-RNAs are so influential that they may be the major determinants of the final response.

Therefore, the identification of a miRNA with a predominant effect on the targeted mRNA of genes of interest may be of considerable value in investigating pathophysiology and planning targeted therapy. In that context, anti-miRNA and miRNA-mimics are potentially useful in the treatment of immune-inflammatory disorders. It is noteworthy however that a single miRNA may nonspecifically affect a broad spectrum of target mRNAs, altering an array of cellular pathways. On the other hand, multiple mi-RNAs may only target a single gene. These and other characteristics pose considerable challenges to mi-RNA-based drugs in the treatment of motility disorders (52).

## PHENOTYPIC CHANGES OR REMODELING IN SMOOTH MUSCLES

The physiological regulation of smooth muscle contraction or relaxation is mediated by neurotransmitters, acting on GPCR to increase or decrease levels of intracellular Ca^2+^ concentration ([Ca^2+^]_i_), respectively. An increase in [Ca^2+^]_i_ activates myosin light chain kinase (MLCK), which initiates the phosphorylation of MLC_20_ (p-MLC_20_), promoting an interaction between actin and myosin as well as cross-bridge cycling, responsible for the commencement of smooth muscle contraction (53). The process of dephosphorylation of p-MLC_20_ by an active myosin light chain phosphatase (MLCP), especially using the regulatory subunit (MYPT1) (54–56), causes the disengagement of interaction between the actin-myosin myofilaments and cross-bridge cycling, resulting in the smooth muscle relaxation, and a short-lasting (phasic) contraction. Conversely, inhibition of MLCP by the specific kinases, e.g., RhoA/ROCK (57), which prolongs p-MLC_20_, results in sustained smooth muscle contraction, the main cause of Ca^2+^ “independent” (tonic) contraction of the smooth muscle phenotype (58, 59).

In response to mechanical stress, smooth muscles secrete a plethora of molecules that regulate their development, differentiation, and growth. The secretory response may determine the development of either non-contractile “secretory” or “contractile” phenotypes (19). The development of a contractile phenotype may not occur in the absence of either mechanical stress, or RhoA/ROCK signaling (60, 61).

Products of MC-associated immune inflammation activate GPCR to elicit contractile responses and activate RTK to induce genetic changes in the smooth muscle. These changes are responsible for the functional (either hypercontractile or hypocontractile) and structural (either hypertrophic, proliferative, or atrophic) phenotypic variants. Therefore, depending on the mediators released, MCs may produce different functional and structural changes in the smooth muscles (62, 63).

## SMOOTH MUSCLE HYPERCONTRACTILITY

Smooth muscle hypercontractility is defined as an enhanced muscle contraction in response to contractile agonists shown by the shift in the dose-response curve towards left, and by an increase in the *V*_max_, beyond the physiological limits (64–66).

Products of MCs (such as leukotrienes, tryptase, TNFα, IL-13, and IL-4 but neither IL-5 nor IL-17A) cause smooth muscle contraction and hypercontractility. The hypercontractility is associated with an elevated activity and expression of MLCK and other contractile proteins, and in the cytoskeleton remodeling proteins (67, 68). RhoA/ROCK plays a key role in the organization of the smooth muscle, nonmuscle cytoskeleton, and in the regulation of transcription factors (68, 69). Interestingly, smooth muscle tonicity and hypercontractility is negatively regulated by miR-133a via downregulation of RhoA/ROCK machinery (70, 71).

Nitrergic and β-adrenergic agonists act to elevate intracellular levels of cAMP and cGMP that decrease levels of [Ca^2+^]_i_ and inhibit RhoA/ROCK. Inhibition of RhoA/ROCK signaling increases MLCP producing smooth muscle relaxation (53, 56, 72). In immune inflammation-induced-hypercontractility of the smooth muscle, and impaired cAMP/cGMP signaling may impair relaxation in response to the inhibitory transmitter, nitric oxide, and to β-adrenoceptor agonists. The loss of cGMP signaling in the immune-inflamed smooth muscle in achalasia may contribute to impaired deglutitive inhibition. The loss of cAMP signaling in the smooth muscle in advanced asthma may explain loss of responsiveness to β-adrenoceptor agonists (48, 49, 73).

## SMOOTH MUSCLE HYPERTROPHY

Smooth muscles of viscera undergo a large increase in volume when there is a chronic, partial obstruction impairing the flow of luminal contents (21). In muscle hypertrophy, gross architecture is preserved but cellular details of hypertrophy in different tissues may vary. In intestinal hypertrophy, the SMCs increase in number, speculatively via mitosis. An enlargement of SMCs accounts for most of the muscle hypertrophy. The hypertrophic SMCs are not only larger than the control but also have a different pattern of structural components (21). Hypertrophy may be associated with either hypercontractility or hypocontractility. The mechanisms involved in hypertrophic growth are unknown. One of the mechanisms of smooth muscle hypertrophy has been shown to be via upregulation of cyclin Dβ by inhibition of GSK-3β (74). It is noteworthy that mechanical stretch-upregulated miR-26a, by its direct interaction with the 3'-UTR of the GSK-3β mRNA, downregulates GSK-3β, unleashing cyclin Dβ and causing smooth muscle hypertrophy.

## SMOOTH MUSCLE PROLIFERATION

Smooth muscle proliferation is usually associated with increased migration that involves cytokine and growth factor-mediated activation of p21 Ras proteins, and downstream signaling pathways. The latter include Rac1-activated transcription factors (SP1, CREB, NF-kB), promoting cyclin D1 gene expression and transcription factors (E2Fs), followed by an increase in the synthesis of cycle G1/S transition genes, collectively leading to cell proliferation (75). Remarkably, miR-17 suppresses retinoblastoma (RB) protein expression, leading to upregulation of E2F (critical regulator of cell cycle) and NF-κB p65/miR-17/RB pathway, promoting cell proliferation. Excessive proliferation of smooth muscle has also been associated with vascular stenotic lesions (76).

## SMOOTH MUSCLE ATROPHY

Smooth muscle atrophy may arise under different circumstances (7, 77), including severe oxidative stress with the involvement of macrophages and type 1 polarization, with the release of cytokines IL-1β, TNF-α, and IFN-γ (77).

Recently, Nelson and colleagues (78) described that SMCs in the achalasic lower esophageal sphincter (LES) were atrophic and decreased in size. Their observations suggest that MC-associated immune inflammation may cause smooth muscle hypertrophy above the obstruction, but atrophy at the site of obstruction.

## MECHANICAL STRESS-INDUCED REMODELING OF ESOPHAGEAL MOTILITY DISORDERS

### Eosinophilic Esophagitis

**Figure 3.**
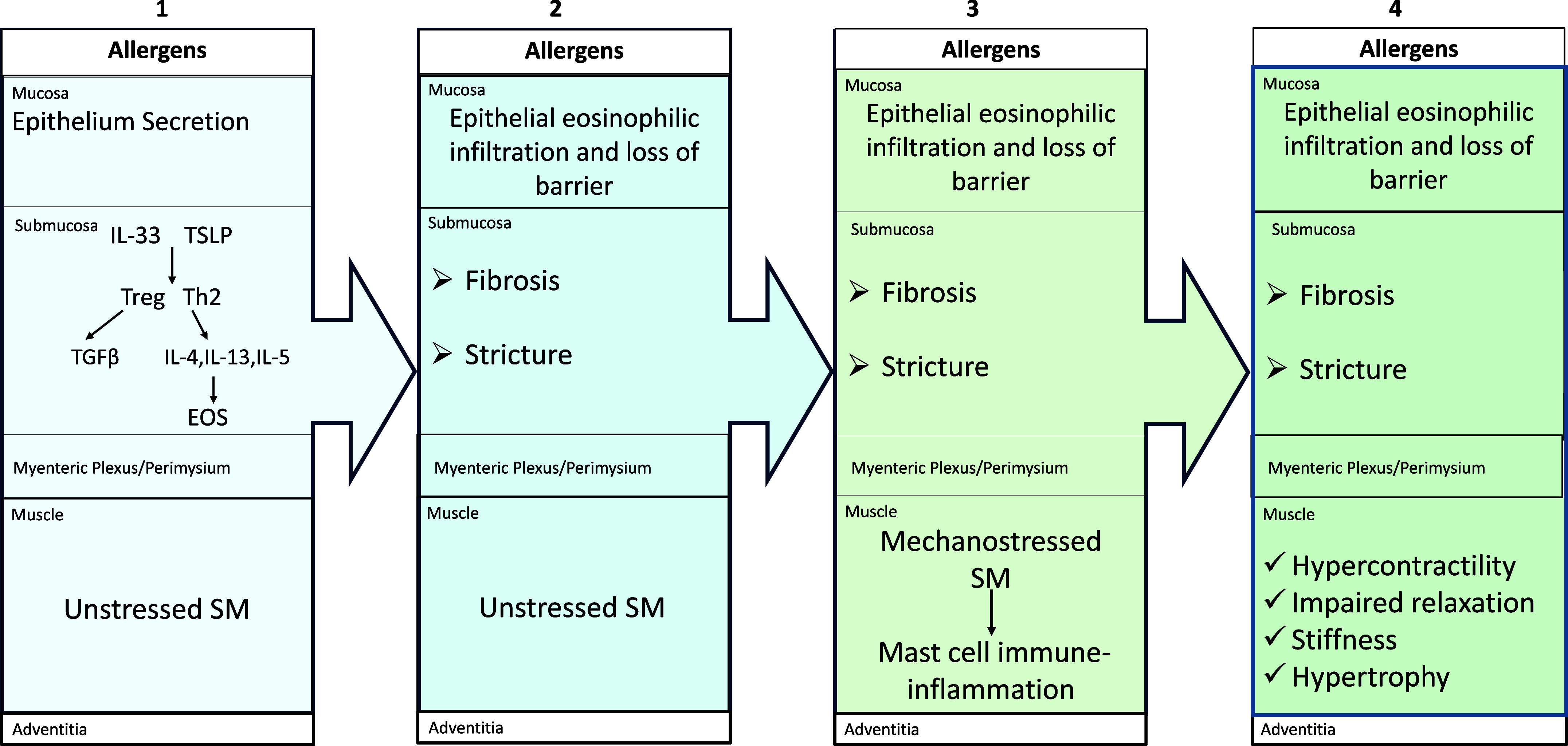
Pathogenesis of smooth muscle hypertrophy in eosinophilic esophagitis (EoE). EoE occurs due to the release of cytokines by the allergy-damaged epithelium; activation of Treg and TH2 cells, release of TGF-β1, IL-4, IL-13, and IL5 and by the recruitment of eosinophils (EOS) (*box 1*). This leads to the development of fibrosis, and submucosal stricture (*box 2*), causing obstruction to the esophageal bolus transport. Thus, mechanostressed smooth muscles secrete chemoattractants, adherents like CADM1, and various stimulants that recruit and microlocalize mast cells to smooth muscle bundles, stimulating them to secrete mediators that act in a paracrine fashion on the smooth muscle to transcribe contractile proteins (*box 3*). This leads to functional and structural changes, such as hypercontractility and hypertrophy (*box 4*).

Eosinophilic esophagitis (EoE) is characterized by the infiltration of: *1*) esophageal mucosa and submucosa with eosinophils and the markers of MCs; and *2*) muscularis propria by prominent MCs (79–81). Figure 3 summarizes the progression of EoE. The latter is initiated by an allergic mucosal injury, which releases allergic inflammation promoters such as IL-33 (a member of the IL-1 cytokine family). These promoters then activate Treg and TH2 cells. The mucosa also has increased expression of transcripts for tryptase, carboxypeptidase, and c-kit ligand, suggesting a role of MCs in the earlier stages of the disease (80). Treg cells release TGF-β1, whereas TH2 cells release IL-4, IL-5, and IL-13. IL-5 increases bone marrow production of eosinophils. IL-4 and IL-13 stimulate mucosal cells to secrete CC motif chemokine 26 (CCL26) that recruits eosinophils to the mucosa, causing eosinophil infiltration. The presence of TGF-β1, IL-4, IL-13, and eosinophil may cause increased epithelial permeability, epithelial-mesenchymal differentiation, and submucosal fibrosis (81). These events then cause loss of distensibility and ring-like stricture, resulting in stressful bolus transport. In motility studies, subepithelial fibrosis accounts for the early pressurization after swallowing and reduced peristaltic contractions (82). The mechanostressed smooth muscles release alarmins that recruit and microlocalize MCs with the smooth muscle. This allows smooth muscle-driven, allergen-independent chronic MC activation, and paracrine release of mediators (such as tryptase, IL-4, and IL-13), leading to MC infiltration. Following this, tryptase, IL-4, IL-13 and TGFβ1 (but not IL-5), released by the MCs cause hypertrophy and hypercontractility of the smooth muscle (79, 83, 84).

## ACHALASIA AND RELATED DISORDERS

These disorders include various versions of achalasia with a common feature of impaired LES relaxation and contractile state of the esophageal body. These include classical achalasia and vigorous achalasia, (types 1, 2, and 3 achalasia, respectively, in high-resolution manometry). Classical achalasia has non-peristaltic, weak, and usually absent contractions. On high-resolution manometry, they are called type 1 and type 2, based on the findings of failed peristalsis and pan esophageal pressurization in response to a swallow. However, there is no difference in the clinical relevance of the two types (3). Vigorous achalasia is characterized by nonperistaltic, high amplitude, long duration, and often repetitive contractions in the esophageal body, and impaired LES relaxation in response to swallowing. When the LES relaxation is normal, but the esophageal body shows nonperistaltic, large repetitive contraction, this is called diffuse esophageal spasm. These disorders may have overlapping features and progress to classical achalasia wherein the hypertonic smooth muscle becomes hypocontractile.

**Figure 4.**
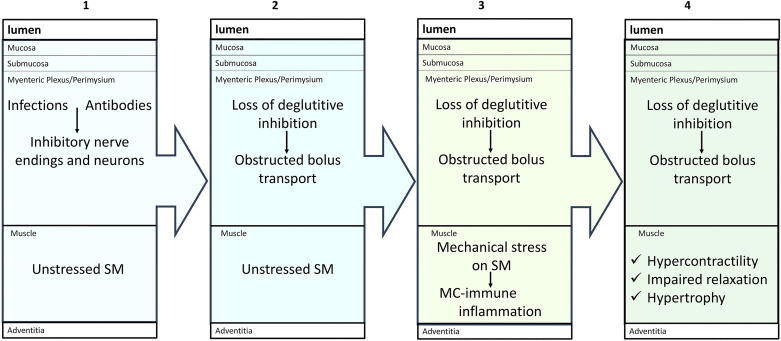
Pathogenesis of hypertrophy, and hypercontractility in achalasia like disorders. Achalasia-like disorders result from the loss of the inhibitory neurotransmission (*box 1*), which may occur due to parasitism, viral infections, or inclusion bodies. These invasions release mediators, which activate MRGPRX2 receptors on the recruited mast cells to the inhibitory neurons. The loss of inhibitory neurons leads to the loss of deglutitive inhibition, and obstruction of esophageal bolus transport (*box 2*). The latter produce mechanical stress on the smooth muscles, which then secrete chemoattractants, adherents like CADM1, and various stimulants, to recruit and microlocalize mast cells. The stimulation of mast cells then secretes mediators, which act in a paracrine fashion on the smooth muscle to transcribe contractile proteins (*box 3*). These events produce functional and structural changes such as hypercontractility, impaired relaxation, and hypertrophy (*box 4*). However, under different types of mechanoobstruction, mast cells may secrete inhibitory mediators causing muscle hypocontractility and atrophy.

Esophageal motility findings in achalasia-related disorders can be divided into two events (Fig. 4): *1*) a loss of deglutitive inhibition (3, 78, 85, 86); and *2*) structural and functional changes in smooth muscles, including hypertrophy or atrophy, and hypercontractility or hypocontractility (3, 78, 87). The first event in the pathogenesis of achalasia is thought to be the loss of inhibitory neuromuscular transmission involving nitric oxide and vasoactive intestinal peptide (VIP) (88, 89). Many causes of loss of inhibitory neurotransmission have been proposed, including the loss of interstitial cells of Cajal (ICC), which may transduce neural signals to the smooth muscles. However, this cause is unlikely because: *1*) loss of inhibitory neuromuscular transmission in achalasia precedes the loss of ICC (90); and *2*) while loss of nitrergic inhibitory transmission causes achalasia, and the loss of ICC in WW/^v^ mutant mice causes hypotensive LES (91). Goldblum and colleagues (92) reported that in achalasia, inhibitory motor nerve fibers, or the myenteric neurons are primarily affected. The loss of inhibitory neuromuscular transmission may also be due to immune inflammation (11, 93). Furthermore, there is growing evidence that MCs may invade myenteric VIP/NO neurons that are already damaged by Lewi body-like inclusions (94), amyloid plaques (95), or *Trypanosoma Cruzi* infection (96).

Recently, herpes simplex virus (HSV)-mediated immune inflammation is proposed to be involved in the development and progression of achalasia. HSV1-derived hsv1-miRNAs that downregulate autophagy-related ATG16L1 gene (which upregulates IL1β), have been identified in biopsy samples of the LES during peroral endoscopic myotomy (POEM) for esophageal achalasia (97). The neuronal inclusions and infections may activate microglia via several receptors, including histocompatibility complex II, which confers susceptibility to idiopathic achalasia (98). Neuronal infection or an allergic response may lead to the production of IgE antibodies, which bind with FcɛR1, causing MC recruitment, degranulation (99), and neuronal damage (100). Nelson and colleagues (7) have shown that in achalasic LES, the degranulating MCs are located in the perimysium, which represents the myenteric plexus (101). The degranulating product is rich in tryptase, which may be involved in neural degeneration (7). This signaling pathway of MC-associated immune damage is akin to nonallergic virus-associated asthma (102). The reason why inhibitory signaling is selectively targeted has been discussed but not well understood.

The second event in the pathogenesis of achalasia is changes in structure and function in the smooth muscles, which include smooth muscle hypertrophy with hypercontractility above the LES smooth muscle, and atrophy at the LES (7). Peristaltic contraction is an amalgam of cholinergic and noncholinergic “rebound” contraction (103). The inhibitory and excitatory neurotransmissions occur sequentially but do not overlap. Therefore, smooth muscle changes cannot be explained by the loss of inhibition and unopposed action of cholinergic activity. Smooth muscle hypercontractility and hypertrophy above the LES are attributed to the increased workload on the smooth muscles to transport a food bolus. This remodeling may represent mechanotransduction and MC-mediated immune inflammation, as in experimental esophageal obstruction (25). MC inflammation also causes muscle rigidity due to impaired myogenic relaxation (78). In advanced achalasia, however, smooth muscle hypercontractility is converted to hypocontractility. This may be due to an increase in the secretion of IL1 and TNFα by the immune cells (14, 104), as in a diabetic stomach (9, 105). In contrast to the smooth muscle hypertrophy above the LES, the LES per se was reported to be atrophic (7). This unexpected finding requires further investigation.

In patients with achalasia, blood levels of both Th1 and Th2 cytokines and other agents (106, 107), including many mi-RNAs, are altered; however, only some of them have been shown to correlate with molecules documented in the pathogenesis of achalasia. These findings suggest that multiple inflammatory processes may be active in achalasia. Interestingly, miR-133 has been associated with RhoA/ROCK signaling, and the smooth muscle contractility and rigidity (70, 71). Inhibition of the miR-200 family is associated with epithelial-to-mesenchymal transition, suppression of NOS, microglia-mediated neurotoxicity, and neuronal apoptosis (108). In addition, miR-150-5p and miR-362-5p upregulate gene MRVI1, which encodes inositol trisphosphate receptor-associated cGMP-kinase substrate (IRAG). IRAG interacts with PKG1 in the SMCs in NO-dependent relaxation (109). Remarkably, downregulation of miR-200c-3p contributes to achalasia disease by targeting the PRKG1 gene (110). However, further studies are needed to define the role of miRNAs in the diagnosis of and potential therapy for achalasia.

## HYPERCONTRACTILE (NUTCRACKER, JACKHAMMER) ESOPHAGUS

In 1979, Benjamin et al. (111) described cases with chest pain or dysphagia who, on manometry, had high amplitude, peristaltic esophageal contraction. These patients were thought to have no other cause of their symptoms. Although such cases had been described before, these colleagues used low compliance pneumohydraulic perfusion manometry to accurately record the contractile pressures. Subsequently, Castell and colleagues nicknamed this condition as nutcracker esophagus. This group of patients was distinguished from diffuse esophageal spasm by nonperistaltic, repetitive, large amplitude contractions to a swallow that were due to the loss of inhibitory neurotransmission. The cause of chest pain in nutcracker esophagus was assumed to be because of larger-than-normal contraction waves, which did not correlate with the symptoms (112). However, the cause of dysphagia in peristaltic hypercontractile esophagus remained unexplained. In contrast, diffuse esophageal spasm, difficult bolus transport and smooth muscle hypercontractility explained the symptoms of dysphagia as well as chest pain.

High-resolution manometry (HRM) was developed to simplify and bring uniformity in the manometric evaluation of motility disorders in different clinical manometry laboratories. In HRM studies, cases of peristaltic as well as nonperistaltic (diffuse esophageal spams) were all lumped together. This motility pattern of repetitive contractions was called Jackhammer esophagus. To simplify the assessment of contraction, the amplitude and duration of esophageal contractions in the distal esophagus in response to a swallow were integrated to obtain a value called contractile integral. However, the rationale for the use of this parameter is unclear, and the contractile integral value to separate normal versus symptomatic patients remains unknown (113, 114). Hong et al. (115) classified Jackhammer esophagus into classical and spastic subtypes based on the distal latencies. However, manometric patterns, whether repetitive or a single large esophageal contraction to swallow, are sometimes grouped under the term hypercontractile esophagus.

From a pathophysiological standpoint, the nonperistaltic nature of the spastic Jackhammer and diffuse esophageal spasm are due to the underlying loss of inhibitory neurotransmission (1). The loss of inhibitory transmission because of the resistance to esophageal bolus transport may produce smooth muscle stress, leading to immune inflammation and thus smooth muscle hypertrophy. It has been reported that cases diagnosed as hypercontractile esophagus have an impaired inhibitory neurotransmission (113, 114, 116–119). The repetitive contractions may represent isolated contraction peaks of individual muscle segments with disruption of cytoskeletal connectivity between the muscle bundles (120).

The peristaltic hypercontractile esophagus may be secondary to mechanical obstruction. Notably, an index case of peristaltic hypercontractile esophagus described by Benjamin and colleagues (111) had a long history of pyrosis, intermittent dysphagia, a hiatal hernia, and esophageal stricture. Muscle thickening is quite common in various primary disorders that cause dysphagia or GERD. Interestingly, it has been shown that experimental partial mechanical obstruction results in smooth muscle hypercontractility and hypertrophy. The muscle hypertrophy and hyperplasia secondary to obstruction may be reversible after the removal of the constricting band (119). Mittal et al. (121) have reported that muscle hypertrophy is a common finding in patients presenting with dysphagia. In such situations, it becomes difficult to determine whether dysphagia is due to overlooked, subtle esophageal constriction. In healthy adults with a Schatzki ring, esophageal lumen diameter of >25 mm ensures the absence of esophageal obstruction (122). Therefore, it is important to document a high degree of luminal patency before excluding mechanotransduction-induced muscle hypertrophy.

Diagnosis of the exact nature of muscle hypertrophy requires muscle biopsies. Unfortunately, systematic muscle biopsy studies examining this issue have not been performed. However, limited case reports of nutcracker esophagus from Japan and China using muscle biopsies had eosinophilic infiltration in the muscle layers (123–127). Some of these patients had high levels of immunoglobulin E (IgE) and responded to steroid treatment (123). Patients with eosinophilic myositis were not associated with eosinophilic mucosal esophagitis (128), except in one case (126). Whether these cases arise from factors other than mechanical stress requires further investigation. Eosinophilic infiltration of the esophagus may take several distinct patterns with associated clinical manifestations, such as eosinophilic mucosal esophagitis, eosinophilic myositis, and eosinophilic achalasia (129). These observations suggest that all cases of esophageal smooth hypertrophy and nutcracker esophagus are due to immune inflammation, but their ultimate underlying cause remains to be determined.

## INEFFECTIVE ESOPHAGEAL MOTILITY AND REDUCED MUSCLE CONTRACTILITY

The term ineffective esophageal motility (IEM) is arbitrarily defined as weak peristaltic contractions associated with the hypotensive LES, which is unable to prevent the reflux of gastric contents into the esophagus. In addition, weak esophageal peristaltic contractions are unable to propel the food bolus or return the refluxed gastric contents back to the stomach. Hypotensive (incompetent) LES may be a compelling cause of GERD and its complications. GERD has an estimated worldwide prevalence of 8%–33%, involving all age groups and all genders (130). Mucosal injury caused by GERD, a prevalent esophageal disorder, may either induce a mild eosinophilic response representing a mild form of EoE (131) or cause long-lasting mucosal damage involving cytokines (132). Notably, GERD associated with Barrett’s esophagus may be further complicated by peptic ulcer and stricture of the esophagus. (133, 134).

Smooth muscle atrophy also occurs in the LES in achalasia (7). Reduced smooth muscle contractions with muscle hypertrophy may also occur above in the esophageal body in advanced stages of achalasia or mechanical esophageal obstruction. Although impaired smooth muscle contractility is a major cause of esophageal motility disorders, its underlying cause remains poorly investigated.

One of the causes of IEM is systemic sclerosis, wherein smooth muscle contractility to cholinergic agents is reduced due to increased antibodies circulating against muscarinic receptors (135). However, other factors may also be involved in smooth muscle atrophy without fibrosis or vascular lesions in scleroderma. In contrast to smooth muscle atrophy, the skin has increased fibrosis. Electron microscopy of the gastric wall shows scant elastic and collagen fibers around the atrophic SMCs without assembly in bundles (136).

Atrophy and senescence may result from suppression of SMC differentiation to contractile type, causing apoptosis. In addition, vascular ischemia may cause severe oxidative stress, leading to the recruitment of lymphocytes, macrophages, and type 1 polarization, releasing cytokines IL-1β, TNF-α, and IFN-γ that induce SMC apoptosis (77). Remarkably, the upregulation of miR-200 and miR-205 associated with the smooth muscle atrophy and senescence, raises the possibility of anti-miRs-200 and 205 as potential candidates for the treatment of hypotensive esophageal motility in systemic sclerosis (137).

In conditions other than scleroderma, immune inflammation may also occur in cases of IEM. For example, in the LES, the site of obstruction in achalasia, degranulating MC infiltration has been associated with atrophic smooth muscle (7). This contrasts with reported smooth muscle hypertrophy in the esophagus above the site of obstruction. Reduced smooth muscle contractions with muscle hypertrophy may also occur above the site of obstruction in the esophageal body in advanced stages of achalasia or mechanical esophageal obstruction. Under certain circumstances, mechanostressed smooth muscles may induce iNOS and COX2 to produce IL-6 and monocyte chemoattractant protein (MCP-1) (14, 138) which in turn release strong smooth muscle inhibitory molecules. Immune inflammatory signaling may transform SMCs from producing excitatory molecules to the inhibitory molecules, with changing local environments (138, 139). Collectively, impaired smooth muscle contractility is a major cause of esophageal motility disorders; however, its underlying cause requires careful investigation.

## CONCLUSIONS

This review provides evidence suggesting that immunological inflammation plays a key role in the pathogenesis of esophageal motility disorders. Although there is a paucity of direct information from the esophagus, parallel studies in other organs, particularly ASM in asthma, provide important guideline models that may be applicable to the esophagus.

Two main immune-inflammatory processes may be involved in esophageal motility disorders. First, the initial immune inflammation is triggered by a variety of external and internal stimuli. In eosinophilic mucosal esophagitis, eosinophil-predominant immune inflammation leads to the formation of esophageal stricture causing mechanical dysphagia. In achalasia, loss of inhibitory nitrergic neurotransmission may occur due to a variety of causes, including immune inflammation involving MCs. Loss of inhibitory transmission causes loss of deglutitive inhibition and motor dysphagia. Dysphagia is a symptom of labored bolus transport that may cause mechanical stress on the smooth muscles. Second, obstructed bolus transport due to increased mechanical and motor activities produces stress on the smooth muscles. The mechanostressed smooth muscles induce a unique immune response involving MCs, as described in the ASM in asthma.

The mechanostressed smooth muscles may secrete a plethora of molecules that attract and microlocalize MCs without eosinophils to the muscle bundles. A “closer than a neural synapse” contact between the SMCs facilitates a direct effect of the cellular secretions on each other to produce a circumscribed effect. MCs are chronically activated by the secretory products (such as IL-33, IL-1α, and ATP) of the stressed SMCs to continuously secrete mediators (like tryptase, leukotrienes, IL4, and IL-13). These mediators then act on the smooth muscles to produce transcriptional changes. This sequence of events may result in smooth muscle hypercontractility and hypertrophy, which further contribute to motor abnormalities.

This review also reveals a paucity of information on the role of immune inflammation on muscle atrophy and hypocontractility, another major cause of esophageal motility disorders. Understanding the pathophysiology of esophageal motility disorders following above laid out novel concepts may advance our approach towards developing rational treatment of esophageal motility disorders.

## GRANTS

This work was supported by the Department of Veterans Affairs, BLR&D, BX02806 (to R.K.G.); Department of Veterans Affairs, Middleton Award (to R.K.G.); National Institute of Diabetes and Digestive and Kidney Diseases Grant R0135385 (to S.R.); and Thomas Jefferson University Institutional Grant (to S.R.).

## DISCLOSURES

No conflicts of interest, financial or otherwise, are declared by the authors.

## AUTHOR CONTRIBUTIONS

R.K.G. and S.R. conceived, designed research, prepared figures; and drafted, edited, revised, and approved final version of the manuscript.

## References

[1] Goyal RK, Chaudhury A. Physiology of normal esophageal motility. J Clin Gastroenterol 42: 610–619, 2008. doi:10.1097/MCG.0b013e31816b444d. 18364578 PMC2728598

[2] Vantrappen GR, Hellemans JJ. Diseases of the Esophagus. Berlin-Heidelberg, New York: Springer Verlag, 1974.

[3] Patel DA, Yadlapati R, Vaezi MF. Esophageal motility disorders: Current approach to diagnostics and therapeutics. Gastroenterology 162: 1617–1634, 2022. doi:10.1053/j.gastro.2021.12.289. 35227779 PMC9405585

[4] Yip JLK, Balasuriya GK, Spencer SJ, Hill-Yardin EL. The role of intestinal macrophages in gastrointestinal homeostasis: Heterogeneity and implications in disease. Cell Mol Gastroenterol Hepatol 12: 1701–1718, 2021. doi:10.1016/j.jcmgh.2021.08.021. 34506953 PMC8551786

[5] Knowles CH, Lindberg G, Panza E, De Giorgio R. New perspectives in the diagnosis and management of enteric neuropathies. Nat Rev Gastroenterol Hepatol 10: 206–218, 2013. doi:10.1038/nrgastro.2013.18. 23399525

[6] O'Shea KM, Aceves SS, Dellon ES, Gupta SK, Spergel JM, Furuta GT, Rothenberg ME. Pathophysiology of eosinophilic esophagitis. Gastroenterology 154: 333–345, 2018. doi:10.1053/j.gastro.2017.06.065. 28757265 PMC5787048

[7] Nelson M, Zhang X, Genta RM, Turner K, Podgaetz E, Paris S, Cardenas J, Gu J, Leeds S, Ward M, Nguyen A, Konda V, Furuta GT, Pan Z, Souza RF, Spechler SJ. Lower esophageal sphincter muscle of patients with achalasia exhibits profound mast cell degranulation. Neurogastroenterol Motil 33: e14055, 2021. doi:10.1111/nmo.14055. 33280206

[8] Shea-Donohue T, Notari L, Sun R, Zhao A. Mechanisms of smooth muscle responses to inflammation. Neurogastroenterol Motil 24: 802–811, 2012. doi:10.1111/j.1365-2982.2012.01986.x. 22908862 PMC4068333

[9] Cipriani G, Gibbons SJ, Miller KE, Yang DS, Terhaar ML, Eisenman ST, Ördög T, Linden DR, Gajdos GB, Szurszewski JH, Farrugia G. Change in populations of macrophages promotes development of delayed gastric emptying in mice. Gastroenterology 154: 2122–2136.e12, 2018. doi:10.1053/j.gastro.2018.02.027. 29501441 PMC5985210

[10] Hayashi Y, Toyomasu Y, Saravanaperumal SA, Bardsley MR, Smestad JA, Lorincz A, Eisenman ST, Cipriani G, Nelson Holte MH, Al Khazal FJ, Syed SA, Gajdos GB, Choi KM, Stoltz GJ, Miller KE, Kendrick ML, Rubin BP, Gibbons SJ, Bharucha AE, Linden DR, Maher LJ 3rd, Farrugia G, Ordog T. Hyperglycemia increases interstitial cells of Cajal via MAPK1 and MAPK3 signaling to ETV1 and KIT, leading to rapid gastric emptying. Gastroenterology 153: 521–535.e20, 2017. doi:10.1053/j.gastro.2017.04.020. 28438610 PMC5526732

[11] Goyal RK, Chaudhury A. Pathogenesis of achalasia: lessons from mutant mice. Gastroenterology 139: 1086–1090, 2010. doi:10.1053/j.gastro.2010.08.013. 20800108

[12] Davis MJ, Earley S, Li YS, Chien S. Vascular mechanotransduction. Physiol Rev 103: 1247–1421, 2023. doi:10.1152/physrev.00053.2021. 36603156 PMC9942936

[13] Collins SM, Khan I, Vallance B, Hogaboam C, Barbara G. Role of smooth muscle in intestinal inflammation. Can J Gastroenterol 10: 249–253, 1996. doi:10.1155/1996/570782.

[14] Li F, Lin YM, Sarna SK, Shi XZ. Cellular mechanism of mechanotranscription in colonic smooth muscle cells. Am J Physiol Gastrointest Liver Physiol 303: G646–G656, 2012. doi:10.1152/ajpgi.00440.2011. 22700825 PMC3468553

[15] Bradding P. Mechanisms of mast cell activation in severe asthma: beyond IgE. Am J Respir Crit Care Med 205: 375–377, 2022. doi:10.1164/rccm.202110-2322ED. 34856107 PMC8886944

[16] Mischopoulou M, D'Ambrosio M, Bigagli E, Luceri C, Farrugia G, Cipriani G. Role of macrophages and mast cells as key players in the maintenance of gastrointestinal smooth muscle homeostasis and disease. Cell Mol Gastroenterol Hepatol 13: 1849–1862, 2022. doi:10.1016/j.jcmgh.2022.02.017. 35245688 PMC9123576

[17] Iskratsch T, Wolfenson H, Sheetz MP. Appreciating force and shape—the rise of mechanotransduction in cell biology. Nat Rev Mol Cell Biol 15: 825–833, 2014. doi:10.1038/nrm3903. 25355507 PMC9339222

[18] Joshi V, Strege PR, Farrugia G, Beyder A. Mechanotransduction in gastrointestinal smooth muscle cells: role of mechanosensitive ion channels. Am J Physiol Gastrointest Liver Physiol 320: G897–G906, 2021. doi:10.1152/ajpgi.00481.2020. 33729004 PMC8202201

[19] Ye GJ, Nesmith AP, Parker KK. The role of mechanotransduction on vascular smooth muscle myocytes' [corrected] cytoskeleton and contractile function. Anat Rec (Hoboken) 297: 1758–1769, 2014 [Erratum in Anat Rec (Hoboken) 298: 637, 2015]. doi:10.1002/ar.22983. 25125187 PMC4863956

[20] Retailleau K, Arhatte M, Demolombe S, Peyronnet R, Baudrie V, Jodar M, Bourreau J, Henrion D, Offermanns S, Nakamura F, Feng Y, Patel A, Duprat F, Honoré E. Arterial myogenic activation through smooth muscle filamin A. Cell Rep 14: 2050–2058, 2016. doi:10.1016/j.celrep.2016.02.019. 26923587

[21] Gabella G. Hypertrophy of visceral smooth muscle. Anat Embryol (Berl) 182: 409–424, 1990. doi:10.1007/bf00178906. 2291488

[22] Mittal RK, Ren J, McCallum RW, Shaffer HA Jr, Sluss J. Modulation of feline esophageal contractions by bolus volume and outflow obstruction. Am J Physiol 258: G208–G215, 1990. doi:10.1152/ajpgi.1990.258.2.G208. 2305886

[23] Tung HN, Schulze-Delrieu K, Shirazi S, Noel S, Xia Q, Cue K. Hypertrophic smooth muscle in the partially obstructed opossum esophagus. The model: histological and ultrastructural observations. Gastroenterology 100: 853–864, 1991. doi:10.1016/0016-5085(91)90256-k. 2001825

[24] Lu C, Schulze-Delrieu K, Shirazi S, Cram M, Raab J. Dynamic imaging of obstructed opossum esophagus. From altered load to altered contractility. Dig Dis Sci 39: 1377–1388, 1994. doi:10.1007/bf02088037. 8026246

[25] Tung HN, Schulze-Delrieu K, Shirazi S. Infiltration of hypertrophic esophageal smooth muscle by mast cells and basophils. J Submicrosc Cytol Pathol 25: 93–102, 1993. 8462073

[26] Shi XZ, Lin YM, Hegde S. Novel insights into the mechanisms of abdominal pain in obstructive bowel disorders. Front Integr Neurosci 12: 23, 2018. doi:10.3389/fnint.2018.00023. 29937720 PMC6002527

[27] Tung HN, Shirazi S, Schulze-Delrieu K, Brown K. Morphological changes of myenteric neurons in the partially obstructed opossum esophagus. J Submicrosc Cytol Pathol 25: 357–363, 1993. 8402535

[28] Conklin JL, Du CA, Schulze-Delrieu K, Shirazi S. Hypertrophic smooth muscle in the partially obstructed opossum esophagus. Excitability and electrophysiological properties. Gastroenterology 101: 657–663, 1991. doi:10.1016/0016-5085(91)90522-m. 1860630

[29] Vodenicharov A. Structure and function of smooth muscle with special reference to mast cells. In: Current Basic And Pathological Approaches To The Function Of Muscle Cells And Tissues - From Molecules To Humans, edited by Sugi H. INTECH, Open Science, Open Mind, 2012; Tokyo University, p. 1–412.

[30] Bradding P, Walls AF, Holgate ST. The role of the mast cell in the pathophysiology of asthma. J Allergy Clin Immunol 117: 1277–1284, 2006. doi:10.1016/j.jaci.2006.02.039. 16750987

[31] Lichtenstein LM, Bochner BS. The role of basophils in asthma. Ann N Y Acad Sci 629: 48–61, 1991. doi:10.1111/j.1749-6632.1991.tb37960.x. 1952578

[32] Brightling CE, Bradding P, Symon FA, Holgate ST, Wardlaw AJ, Pavord ID. Mast-cell infiltration of airway smooth muscle in asthma. N Engl J Med 346: 1699–1705, 2002. doi:10.1056/NEJMoa012705. 12037149

[33] Nasser SM, Pulimood TB. Allergens and thunderstorm asthma. Curr Allergy Asthma Rep 9: 384–390, 2009. doi:10.1007/s11882-009-0056-8. 19671382

[34] Bradding P, Arthur G. Mast cells in asthma–state of the art. Clin Exp Allergy 46: 194–263, 2016. doi:10.1111/cea.12675. 26567481

[35] Dvorak AM, Tepper RI, Weller PF, Morgan ES, Estrella P, Monahan-Earley RA, Galli SJ. Piecemeal degranulation of mast cells in the inflammatory eyelid lesions of interleukin-4 transgenic mice. Evidence of mast cell histamine release in vivo by diamine oxidase-gold enzyme-affinity ultrastructural cytochemistry. Blood 83: 3600–3612, 1994. doi:10.1182/blood.V83.12.3600.3600. 7515717

[36] Goyal RK, Chaudhury A. Structure activity relationship of synaptic and junctional neurotransmission. Auton Neurosci 176: 11–31, 2013. doi:10.1016/j.autneu.2013.02.012. 23535140 PMC3677731

[37] Gaudenzio N, Sibilano R, Marichal T, Starkl P, Reber LL, Cenac N, McNeil BD, Dong X, Hernandez JD, Sagi-Eisenberg R, Hammel I, Roers A, Valitutti S, Tsai M, Espinosa E, Galli SJ. Different activation signals induce distinct mast cell degranulation strategies. J Clin Invest 126: 3981–3998, 2016. doi:10.1172/jci85538. 27643442 PMC5096814

[38] Ali H. Mas-related G protein coupled receptor-X2: a potential new target for modulating mast cell-mediated allergic and inflammatory diseases. J Immunobiol 1: 115, 2016. doi:10.4172/2476-1966.1000115. 28090599 PMC5233413

[39] Porebski G, Kwiecien K, Pawica M, Kwitniewski M. Mas-related G protein-coupled receptor-X2 (MRGPRX2) in drug hypersensitivity reactions. Front Immunol 9: 3027, 2018. doi:10.3389/fimmu.2018.03027. 30619367 PMC6306423

[40] Bax HJ, Keeble AH, Gould HJ. Cytokinergic IgE action in mast cell activation. Front Immunol 3: 229, 2012. doi:10.3389/fimmu.2012.00229. 22888332 PMC3412263

[41] Espinosa-Riquer ZP, Segura-Villalobos D, Ramírez-Moreno IG, Pérez Rodríguez MJ, Lamas M, Gonzalez-Espinosa C. Signal transduction pathways activated by innate immunity in mast cells: translating sensing of changes into specific responses. Cells 9: 2411, 2020. doi:10.3390/cells9112411. 33158024 PMC7693401

[42] Ito A, Hagiyama M, Oonuma J. Nerve-mast cell and smooth muscle-mast cell interaction mediated by cell adhesion molecule-1, CADM1. J Smooth Muscle Res 44: 83–93, 2008. doi:10.1540/jsmr.44.83. 18552455

[43] Moiseeva EP, Roach KM, Leyland ML, Bradding P. CADM1 is a key receptor mediating human mast cell adhesion to human lung fibroblasts and airway smooth muscle cells. PLoS One 8: e61579, 2013. doi:10.1371/journal.pone.0061579. 23620770 PMC3631237

[44] Ribatti D, Crivellato E. Mast cell ontogeny: an historical overview. Immunol Lett 159: 11–14, 2014. doi:10.1016/j.imlet.2014.02.003. 24534641

[45] Cruse G, Bradding P. Mast cells in airway diseases and interstitial lung disease. Eur J Pharmacol 778: 125–138, 2016. doi:10.1016/j.ejphar.2015.04.046. 25959386 PMC4637266

[46] Hollins F, Kaur D, Yang W, Cruse G, Saunders R, Sutcliffe A, Berger P, Ito A, Brightling CE, Bradding P. Human airway smooth muscle promotes human lung mast cell survival, proliferation, and constitutive activation: cooperative roles for CADM1, stem cell factor, and IL-6. J Immunol 181: 2772–2780, 2008. doi:10.4049/jimmunol.181.4.2772. 18684968 PMC3992374

[47] Virk H, Arthur G, Bradding P. Mast cells and their activation in lung disease. Transl Res 174: 60–76, 2016. doi:10.1016/j.trsl.2016.01.005. 26845625

[48] Alzahrani A, Hakeem J, Biddle M, Alhadian F, Hussain A, Khalfaoui L, Roach KM, Tliba O, Bradding P, Amrani Y. Human lung mast cells impair corticosteroid responsiveness in human airway smooth muscle cells. Front Allergy 2: 785100, 2021. doi:10.3389/falgy.2021.785100. 35387008 PMC8974721

[49] Amrani Y, Bradding P. β2-adrenoceptor function in asthma. Adv Immunol 136: 1–28, 2017. doi:10.1016/bs.ai.2017.06.003. 28950943

[50] Hu X, Li J, Fu M, Zhao X, Wang W. The JAK/STAT signaling pathway: from bench to clinic. Sig Transduct Target Ther 6: 402, 2021. doi:10.1038/s41392-021-00791-1. 34824210 PMC8617206

[51] O'Brien J, Hayder H, Zayed Y, Peng C. Overview of microrna biogenesis, mechanisms of actions, and circulation. Front Endocrinol (Lausanne) 9: 402, 2018. doi:10.3389/fendo.2018.00402. 30123182 PMC6085463

[52] Diener C, Keller A, Meese E. Emerging concepts of miRNA therapeutics: from cells to clinic. Trends Genet 38: 613–626, 2022. doi:10.1016/j.tig.2022.02.006. 35303998

[53] Murthy KS. Signaling for contraction and relaxation in smooth muscle of the gut. Annu Rev Physiol 68: 345–374, 2006. doi:10.1146/annurev.physiol.68.040504.094707. 16460276

[54] Amano M, Kanazawa Y, Kozawa K, Kaibuchi K. Identification of the kinase-substrate recognition interface between MYPT1 and rho-kinase. Biomolecules 12: 159, 2022. doi:10.3390/biom12020159. 35204659 PMC8869655

[55] Rattan S. Smooth muscle-specific myosin phosphatase target subunit 1 (MYPT1): an important piece of the puzzle. Gastroenterology 145: 1494–1495, 2013. doi:10.1053/j.gastro.2013.07.055. 24172015 PMC3888222

[56] Schaub MC. Myosin light chain phosphatase. In: The Comprehensive Pharmacology Reference, edited by Enna S, Bylund DB. New York: Elsevier, 1–3, 2007.

[57] Rattan S, Phillips BR, Maxwell PJt. RhoA/Rho-kinase: pathophysiologic and therapeutic implications in gastrointestinal smooth muscle tone and relaxation. Gastroenterology 138: 13–18.e1-3, 2010. doi:10.1053/j.gastro.2009.11.016. 19931260 PMC5599165

[58] Álvarez-Santos MD, Álvarez-González M, Estrada-Soto S, Bazán-Perkins B. Regulation of myosin light-chain phosphatase activity to generate airway smooth muscle hypercontractility. Front Physiol 11: 701, 2020. doi:10.3389/fphys.2020.00701. 32676037 PMC7333668

[59] Rattan S. Ca^2+^/calmodulin/MLCK pathway initiates, and RhoA/ROCK maintains, the internal anal sphincter smooth muscle tone. Am J Physiol Gastrointest Liver Physiol 312: G63–G66, 2017. doi:10.1152/ajpgi.00370.2016. 27932502 PMC5283903

[60] Sawma T, Shaito A, Najm N, Sidani M, Orekhov A, El-Yazbi AF, Iratni R, Eid AH. Role of RhoA and Rho-associated kinase in phenotypic switching of vascular smooth muscle cells: Implications for vascular function. Atherosclerosis 358: 12–28, 2022. doi:10.1016/j.atherosclerosis.2022.08.012. 36049290

[61] Smith PG, Roy C, Zhang YN, Chauduri S. Mechanical stress increases RhoA activation in airway smooth muscle cells. Am J Respir Cell Mol Biol 28: 436–442, 2003. doi:10.1165/rcmb.4754. 12654632

[62] Carter RJ, Bradding P. The role of mast cells in the structural alterations of the airways as a potential mechanism in the pathogenesis of severe asthma. Curr Pharm Des 17: 685–698, 2011. doi:10.2174/138161211795428975. 21410430

[63] Dilasser F, Rose L, Hassoun D, Klein M, Rousselle M, Brosseau C, Guignabert C, Taillé C, Dombret MC, Di Candia L, Heddebaut N, Bouchaud G, Pretolani M, Magnan A, Loirand G, Sauzeau V. Essential role of smooth muscle Rac1 in severe asthma-associated airway remodelling. Thorax 76: 326–334, 2021. doi:10.1136/thoraxjnl-2020-216271. 33542087 PMC7982925

[64] Ehlert FJ, Sawyer GW, Esqueda EE. Contractile role of M2 and M3 muscarinic receptors in gastrointestinal smooth muscle. Life Sci 64: 387–394, 1999 [Erratum in Life Sci 64: 2535, 1999]. doi:10.1016/s0024-3205(98)00584-0. 10069501

[65] Ijpma G, Panariti A, Lauzon AM, Martin JG. Directional preference of airway smooth muscle mass increase in human asthmatic airways. Am J Physiol Lung Cell Mol Physiol 312: L845–L854, 2017. doi:10.1152/ajplung.00353.2016. 28360113

[66] Yap HM, Israf DA, Harith HH, Tham CL, Sulaiman MR. Crosstalk between signaling pathways involved in the regulation of airway smooth muscle cell hyperplasia. Front Pharmacol 10: 1148, 2019. doi:10.3389/fphar.2019.01148. 31649532 PMC6794426

[67] Ojiaku CA, Yoo EJ, Panettieri RA Jr. Transforming growth factor β1 function in airway remodeling and hyperresponsiveness. The missing link? Am J Respir Cell Mol Biol 56: 432–442, 2017. doi:10.1165/rcmb.2016-0307TR. 27854509 PMC5449515

[68] Sieck GC, Dogan M, Young-Soo H, Osorio Valencia S, Delmotte P. Mechanisms underlying TNFα-induced enhancement of force generation in airway smooth muscle. Physiol Rep 7: e14220, 2019. doi:10.14814/phy2.14220. 31512410 PMC6739507

[69] Jaffe AB, Hall A. Rho GTPases: biochemistry and biology. Annu Rev Cell Dev Biol 21: 247–269, 2005. doi:10.1146/annurev.cellbio.21.020604.150721. 16212495

[70] Singh J, Boopathi E, Addya S, Phillips B, Rigoutsos I, Penn RB, Rattan S. Aging-associated changes in microRNA expression profile of internal anal sphincter smooth muscle: Role of microRNA-133a. Am J Physiol Gastrointest Liver Physiol 311: G964–G973, 2016. doi:10.1152/ajpgi.00290.2016. 27634012 PMC5130548

[71] Chiba Y, Tanabe M, Goto K, Sakai H, Misawa M. Down-regulation of miR-133a contributes to up-regulation of Rhoa in bronchial smooth muscle cells. Am J Respir Crit Care Med 180: 713–719, 2009. doi:10.1164/rccm.200903-0325OC. 19644046

[72] Xiong DJP, Martin JG, Lauzon AM. Airway smooth muscle function in asthma. Front Physiol 13: 993406, 2022. doi:10.3389/fphys.2022.993406. 36277199 PMC9581182

[73] Penn RB, Panettieri RA Jr, Benovic JL. Mechanisms of acute desensitization of the β2AR-adenylyl cyclase pathway in human airway smooth muscle. Am J Respir Cell Mol Biol 19: 338–348, 1998. doi:10.1165/ajrcmb.19.2.3025. 9698608

[74] Mohamed JS, Lopez MA, Boriek AM. Mechanical stretch up-regulates microRNA-26a and induces human airway smooth muscle hypertrophy by suppressing glycogen synthase kinase-3β. J Biol Chem 285: 29336–29347, 2010. doi:10.1074/jbc.M110.101147. 20525681 PMC2937966

[75] Yang D, Sun C, Zhang J, Lin S, Zhao L, Wang L, Lin R, Lv J, Xin S. Proliferation of vascular smooth muscle cells under inflammation is regulated by NF-κB p65/microRNA-17/RB pathway activation. Int J Mol Med 41: 43–50, 2018. doi:10.3892/ijmm.2017.3212. 29115381 PMC5746293

[76] Luo T, Cui S, Bian C, Yu X. Crosstalk between TGF-β/Smad3 and BMP/BMPR2 signaling pathways via miR-17-92 cluster in carotid artery restenosis. Mol Cell Biochem 389: 169–176, 2014. doi:10.1007/s11010-013-1938-6. 24378993 PMC4221161

[77] Geng YJ, Wu Q, Muszynski M, Hansson GK, Libby P. Apoptosis of vascular smooth muscle cells induced by in vitro stimulation with interferon-γ, tumor necrosis factor-α, and interleukin-1β. Arterioscler Thromb Vasc Biol 16: 19–27, 1996. doi:10.1161/01.atv.16.1.19. 8548421

[78] Nelson M, Zhang X, Pan Z, Spechler SJ, Souza RF. Mast cell effects on esophageal smooth muscle and their potential role in eosinophilic esophagitis and achalasia. Am J Physiol Gastrointest Liver Physiol 320: G319–G327, 2021. doi:10.1152/ajpgi.00290.2020. 33355505

[79] Aceves SS, Chen D, Newbury RO, Dohil R, Bastian JF, Broide DH. Mast cells infiltrate the esophageal smooth muscle in patients with eosinophilic esophagitis, express TGF-β1, and increase esophageal smooth muscle contraction. J Allergy Clin Immunol 126: 1198–1204.e4, 2010. doi:10.1016/j.jaci.2010.08.050. 21047675

[80] Abonia JP, Blanchard C, Butz BB, Rainey HF, Collins MH, Stringer K, Putnam PE, Rothenberg ME. Involvement of mast cells in eosinophilic esophagitis. J Allergy Clin Immunol 126: 140–149, 2010. doi:10.1016/j.jaci.2010.04.009. 20538331 PMC2902643

[81] Dellon ES, Hirano I. Epidemiology and natural history of eosinophilic esophagitis. Gastroenterology 154: 319–332.e3, 2018. doi:10.1053/j.gastro.2017.06.067. 28774845 PMC5794619

[82] Roman S, Hirano I, Kwiatek MA, Gonsalves N, Chen J, Kahrilas PJ, Pandolfino JE. Manometric features of eosinophilic esophagitis in esophageal pressure topography. Neurogastroenterol Motil 23: 208–214.e111, 2011. doi:10.1111/j.1365-2982.2010.01633.x. 21091849 PMC3036777

[83] Mavi P, Rajavelu P, Rayapudi M, Paul RJ, Mishra A. Esophageal functional impairments in experimental eosinophilic esophagitis. Am J Physiol Gastrointest Liver Physiol 302: G1347–G1355, 2012. doi:10.1152/ajpgi.00013.2012. 22361731 PMC3378164

[84] Niranjan R, Mavi P, Rayapudi M, Dynda S, Mishra A. Pathogenic role of mast cells in experimental eosinophilic esophagitis. Am J Physiol Gastrointest Liver Physiol 304: G1087–G1094, 2013. doi:10.1152/ajpgi.00070.2013. 23599040 PMC3680716

[85] Yamato S, Spechler SJ, Goyal RK. Role of nitric oxide in esophageal peristalsis in the opossum. Gastroenterology 103: 197–204, 1992. doi:10.1016/0016-5085(92)91113-I. 1612326

[86] Sifrim D, Janssens J, Vantrappen G. Failing deglutitive inhibition in primary esophageal motility disorders. Gastroenterology 106: 875–882, 1994. doi:10.1016/0016-5085(94)90745-5. 8143993

[87] Vantrappen G, Janssens J, Hellemans J, Coremans G. Achalasia, diffuse esophageal spasm, and related motility disorders. Gastroenterology 76: 450–457, 1979. 428703

[88] Goyal RK, Rattan S, Said SI. VIP as a possible neurotransmitter of non-cholinergic non-adrenergic inhibitory neurones. Nature 288: 378–380, 1980. doi:10.1038/288378a0. 6107863

[89] Mashimo H, Goyal RK. Lessons from genetically engineered animal models. IV. Nitric oxide synthase gene knockout mice. Am J Physiol Gastrointest Liver Physiol 277: G745–G750, 1999. doi:10.1152/ajpgi.1999.277.4.G745. 10516139

[90] Zarate N, Wang XY, Tougas G, Anvari M, Birch D, Mearin F, Malagelada JR, Huizinga JD. Intramuscular interstitial cells of Cajal associated with mast cells survive nitrergic nerves in achalasia. Neurogastroenterol Motil 18: 556–568, 2006. doi:10.1111/j.1365-2982.2006.00788.x. 16771771

[91] Sivarao DV, Mashimo HL, Thatte HS, Goyal RK. Lower esophageal sphincter is achalasic in nNOS(−/−) and hypotensive in W/W(v) mutant mice. Gastroenterology 121: 34–42, 2001. doi:10.1053/gast.2001.25541. 11438492

[92] Goldblum JR, Rice TW, Richter JE. Histopathologic features in esophagomyotomy specimens from patients with achalasia. Gastroenterology 111: 648–654, 1996. doi:10.1053/gast.1996.v111.pm8780569. 8780569

[93] Clark SB, Rice TW, Tubbs RR, Richter JE, Goldblum JR. The nature of the myenteric infiltrate in achalasia: an immunohistochemical analysis. Am J Surg Pathol 24: 1153–1158, 2000. doi:10.1097/00000478-200008000-00014. 10935657

[94] Qualman SJ, Haupt HM, Yang P, Hamilton SR. Esophageal Lewy bodies associated with ganglion cell loss in achalasia. Similarity to Parkinson's disease. Gastroenterology 87: 848–856, 1984. 6088351

[95] Han S, Kollmer M, Markx D, Claus S, Walther P, Fändrich M. Amyloid plaque structure and cell surface interactions of β-amyloid fibrils revealed by electron tomography. Sci Rep 7: 43577, 2017. doi:10.1038/srep43577. 28240273 PMC5327471

[96] Ricci MF, Béla SR, Moraes MM, Bahia MT, Mazzeti AL, Oliveira ACS, Andrade LO, Radí R, Piacenza L, Arantes RME. Neuronal parasitism, early myenteric neurons depopulation and continuous axonal networking damage as underlying mechanisms of the experimental intestinal Chagas' disease. Front Cell Infect Microbiol 10: 583899, 2020. doi:10.3389/fcimb.2020.583899. 33178632 PMC7597600

[97] Ikebuchi Y, Kanda T, Ikeda H, Yoshida A, Sakaguchi T, Urabe S, Minami H, Nakao K, Kuwamoto S, Inoue H, Isomoto H. Identification of human herpes virus 1 encoded microRNAs in biopsy samples of lower esophageal sphincter muscle during peroral endoscopic myotomy for esophageal achalasia. Dig Endosc 32: 136–142, 2020. doi:10.1111/den.13491. 31325192

[98] Gockel I, Becker J, Wouters MM, Niebisch S, Gockel HR, Hess T et al.Common variants in the HLA-DQ region confer susceptibility to idiopathic achalasia. Nat Genet 46: 901–904, 2014. doi:10.1038/ng.3029. 24997987

[99] Andersson CK, Bergqvist A, Mori M, Mauad T, Bjermer L, Erjefält JS. Mast cell-associated alveolar inflammation in patients with atopic uncontrolled asthma. J Allergy Clin Immunol 127: 905–912.e901-907, 2011. doi:10.1016/j.jaci.2011.01.022. 21388666

[100] Martins PR, Nascimento RD, de Souza Lisboa A, Martinelli PM, d'Ávila Reis D. Neuroimmunopathology of Trypanosoma cruzi-induced megaoesophagus: Is there a role for mast cell proteases? Hum Immunol 75: 302–305, 2014. doi:10.1016/j.humimm.2014.02.003. 24530752

[101] Sengupta A, Paterson WG, Goyal RK. Atypical localization of myenteric neurons in the opossum lower esophageal sphincter. Am J Anat 180: 342–348, 1987. doi:10.1002/aja.1001800404. 3321972

[102] Marshall JS, Portales-Cervantes L, Leong E. Mast cell responses to viruses and pathogen products. Int J Mol Sci 20: 4241, 2019. doi:10.3390/ijms20174241. 31480219 PMC6747121

[103] Crist J, Gidda JS, Goyal RK. Intramural mechanism of esophageal peristalsis: roles of cholinergic and noncholinergic nerves. Proc Natl Acad Sci USA 81: 3595–3599, 1984. doi:10.1073/pnas.81.11.3595. 6587375 PMC345556

[104] Shi XZ. Mechanical regulation of gene expression in gut smooth muscle cells. Front Physiol 8: 1000, 2017. doi:10.3389/fphys.2017.01000. 29259559 PMC5723328

[105] Goyal RK. Gastric emptying abnormalities in diabetes mellitus. N Engl J Med 384: 1742–1751, 2021. doi:10.1056/NEJMra2020927. 33951363

[106] Kanda T, Yoshida A, Ogihara K, Minami H, Yamaguchi N, Ikebuchi Y, Nakao K, Isomoto H. Detection of cytokine storm in patients with achalasia using ELISA. Biomed Rep 15: 62, 2021. doi:10.3892/br.2021.1438. 34113444 PMC8188164

[107] Kanda T, Yoshida A, Ikebuchi Y, Ikeda H, Sakaguchi T, Urabe S, Minami H, Nakao K, Inoue H, Isomoto H. Autophagy-related 16-like 1 is influenced by human herpes virus 1-encoded microRNAs in biopsy samples from the lower esophageal sphincter muscle during per-oral endoscopic myotomy for esophageal achalasia. Biomed Rep 14: 7, 2021. doi:10.3892/br.2020.1383. 33235722 PMC7678595

[108] Palmieri O, Mazza T, Bassotti G, Merla A, Tolone S, Biagini T, Cuttitta A, Bossa F, Martino G, Latiano T, Corritore G, Gioffreda D, Palumbo O, Carella M, Panza A, Andriulli A, Latiano A. microRNA-mRNA network model in patients with achalasia. Neurogastroenterol Motil 32: e13764, 2020. doi:10.1111/nmo.13764. 31773868

[109] Fritsch RM, Saur D, Kurjak M, Oesterle D, Schlossmann J, Geiselhöringer A, Hofmann F, Allescher HD. InsP3R-associated cGMP kinase substrate (IRAG) is essential for nitric oxide-induced inhibition of calcium signaling in human colonic smooth muscle. J Biol Chem 279: 12551–12559, 2004. doi:10.1074/jbc.M313365200. 14729908

[110] Micale L, Fusco C, Nardella G, Palmieri O, Latiano T, Gioffreda D, Tavano F, Panza A, Merla A, Biscaglia G, Gentile M, Cuttitta A, Castori M, Perri F, Latiano A. Downexpression of miR-200c-3p contributes to achalasia disease by targeting the PRKG1 gene. Int J Mol Sci 24: 668, 2022. doi:10.3390/ijms24010668. 36614110 PMC9820813

[111] Benjamin SB, Gerhardt DC, Castell DO. High amplitude, peristaltic esophageal contractions associated with chest pain and/or dysphagia. Gastroenterology 77: 478–483, 1979. 456842

[112] Agrawal A, Hila A, Tutuian R, Mainie I, Castell DO. Clinical relevance of the nutcracker esophagus: suggested revision of criteria for diagnosis. J Clin Gastroenterol 40: 504–509, 2006. doi:10.1097/00004836-200607000-00008. 16825932

[113] Achem SR, Vazquez-Elizondo G, Fass R. Jackhammer esophagus: current concepts and dilemmas. J Clin Gastroenterol 55: 369–379, 2021. doi:10.1097/MCG.0000000000001472. 33337637

[114] Chen JW, Savarino E, Smout A, Xiao Y, de Bortoli N, Yadlapati R, Cock C. Chicago classification update (v4.0): Technical review on diagnostic criteria for hypercontractile esophagus. Neurogastroenterol Motil 33: e14115, 2021. doi:10.1111/nmo.14115. 33666299

[115] Hong YS, Min YW, Rhee PL. Two distinct types of hypercontractile esophagus: classic and spastic Jackhammer. Gut Liver 10: 859–863, 2016. doi:10.5009/gnl15388. 27458179 PMC5003212

[116] Mauro A, Quader F, Tolone S, Savarino E, De Bortoli N, Franchina M, Gyawali CP, Penagini R. Provocative testing in patients with jackhammer esophagus: evidence for altered neural control. Am J Physiol Gastrointest Liver Physiol 316: G397–G403, 2019. doi:10.1152/ajpgi.00342.2018. 30543463

[117] Gawrieh S, Carroll T, Hogan WJ, Soergel KH, Shaker R. Swallow syncope in association with Schatzki ring and hypertensive esophageal peristalsis: report of three cases and review of the literature. Dysphagia 20: 273–277, 2005. doi:10.1007/s00455-005-0024-y. 16633871

[118] Quader F, Mauro A, Savarino E, Tolone S, de Bortoli N, Franchina M, Ghisa M, Edelman K, Jha LK, Penagini R, Gyawali CP. Jackhammer esophagus with and without esophagogastric junction outflow obstruction demonstrates altered neural control resembling type 3 achalasia. Neurogastroenterol Motil 31: e13678, 2019. doi:10.1111/nmo.13678. 31310444

[119] Woo M, Andrews CN, Buresi M. Reversible Jackhammer esophagus in a patient with a gastric band. Neurogastroenterol Motil 31: e13572, 2019. doi:10.1111/nmo.13572. 30843357

[120] Clouse RE, Staiano A, Alrakawi A. Topographic analysis of esophageal double-peaked waves. Gastroenterology 118: 469–476, 2000. doi:10.1016/s0016-5085(00)70252-6. 10702197

[121] Mittal RK, Kassab G, Puckett JL, Liu J. Hypertrophy of the muscularis propria of the lower esophageal sphincter and the body of the esophagus in patients with primary motility disorders of the esophagus. Am J Gastroenterol 98: 1705–1712, 2003. doi:10.1111/j.1572-0241.2003.07587.x. 12907322

[122] Schatzki R. The lower esophageal ring. Long term follow-up of symptomatic and asymptomatic ringS. Am J Roentgenol Radium Ther Nucl Med 90: 805–810, 1963. 14068418

[123] Sato H, Takeuchi M, Takahashi K, Sato Y, Hashimoto S, Mizuno K, Suzuki K, Kobayashi M, Honma T, Inoue H, Terai S. Nutcracker and jackhammer esophagus treatment: a three-case survey, including two novel cases of eosinophilic infiltration into the muscularis propria. Endoscopy 47: 855–857, 2015. doi:10.1055/s-0034-1391985. 25961439

[124] Sato H, Nakajima N, Hasegawa G, Kawata Y, Sato Y, Suzuki K, Honma T, Terai S. Immunohistochemical differentiation of eosinophilic esophageal myositis from eosinophilic esophagitis. J Gastroenterol Hepatol 32: 106–113, 2017. doi:10.1111/jgh.13466. 27262491

[125] Nakajima N, Sato H, Takahashi K, Hasegawa G, Mizuno K, Hashimoto S, Sato Y, Terai S. Muscle layer histopathology and manometry pattern of primary esophageal motility disorders including achalasia. Neurogastroenterology Motil 29: e12968, 2017. doi:10.1111/nmo.12968. 27699951

[126] Tanaka S, Toyonaga T, Kawara F, Watanabe D, Hoshi N, Abe H, Ariyoshi R, Ohara Y, Ishida T, Takao T, Morita Y, Umegaki E. A case of Jackhammer esophagus caused by eosinophilic esophagitis in which per-oral endoscopic myotomy resulted in symptom improvement. Clin J Gastroenterol 11: 377–381, 2018. doi:10.1007/s12328-018-0868-y. 29790076

[127] Tang Y, Xiong W, Yu T, Wang M, Zhang G, Lin L. Eosinophilic esophageal myositis a plausible cause of histological changes of primary jackhammer esophagus: a case report. Am J Gastroenterol 113: 150–152, 2018. doi:10.1038/ajg.2017.433. 29311727

[128] Sato H, Terai S. Eosinophilic esophageal myositis (EoEM) causes jackhammer esophagus, rarely posing a problem in the differential diagnosis of eosinophilic esophagitis. Am J Gastroenterol 113: 1263–1264, 2018. doi:10.1038/s41395-018-0171-z. 29895983

[129] Spechler SJ, Konda V, Souza R. Can eosinophilic esophagitis cause achalasia and other esophageal motility disorders? Am J Gastroenterol 113: 1594–1599, 2018. doi:10.1038/s41395-018-0240-3. 30315308

[130] El-Serag HB, Sweet S, Winchester CC, Dent J. Update on the epidemiology of gastro-oesophageal reflux disease: a systematic review. Gut 63: 871–880, 2014. doi:10.1136/gutjnl-2012-304269. 23853213 PMC4046948

[131] Zhao Y, Ma T, Zou D. Identification of unique transcriptomic signatures and hub genes through rna sequencing and integrated wgcna and ppi network analysis in nonerosive reflux disease. J Inflamm Res 14: 6143–6156, 2021. doi:10.2147/jir.S340452. 34848992 PMC8627320

[132] Dunbar KB, Agoston AT, Odze RD, Huo X, Pham TH, Cipher DJ, Castell DO, Genta RM, Souza RF, Spechler SJ. Association of acute gastroesophageal reflux disease with esophageal histologic changes. JAMA 315: 2104–2112, 2016. doi:10.1001/jama.2016.5657. 27187303 PMC5030713

[133] Chairta P, Nicolaou P, Christodoulou K. Genomic and genetic studies of systemic sclerosis: A systematic review. Hum Immunol 78: 153–165, 2017. doi:10.1016/j.humimm.2016.10.017. 27984087

[134] Avouac J, Fürnrohr BG, Tomcik M, Palumbo K, Zerr P, Horn A, Dees C, Akhmetshina A, Beyer C, Distler O, Schett G, Allanore Y, Distler JH. Inactivation of the transcription factor STAT-4 prevents inflammation-driven fibrosis in animal models of systemic sclerosis. Arthritis Rheum 63: 800–809, 2011. doi:10.1002/art.30171. 21360510

[135] Kumar S, Singh J, Rattan S, DiMarino AJ, Cohen S, Jimenez SA. Review article: pathogenesis and clinical manifestations of gastrointestinal involvement in systemic sclerosis. Aliment Pharmacol Ther 45: 883–898, 2017. doi:10.1111/apt.13963. 28185291 PMC5576448

[136] Treacy WL, Baggenstoss AH, Slocumb CH, Code CF. Scleroderma of the esophagus. A correlation of histologic and physiologic findings. Ann Intern Med 59: 351–356, 1963. doi:10.7326/0003-4819-59-3-351. 14065952

[137] Du M, Espinosa-Diez C, Liu M, Ahmed IA, Mahan S, Wei J, Handen AL, Chan SY, Gomez D. miRNA/mRNA co-profiling identifies the miR-200 family as a central regulator of SMC quiescence. iScience 25: 104169, 2022. doi:10.1016/j.isci.2022.104169. 35465051 PMC9018390

[138] Lin YM, Li F, Shi XZ. Mechanical stress is a pro-inflammatory stimulus in the gut: in vitro, in vivo and ex vivo evidence. PLoS One 9: e106242, 2014. doi:10.1371/journal.pone.0106242. 25180799 PMC4152012

[139] Geesala R, Lin YM, Zhang K, Shi XZ. Targeting mechano-transcription process as therapeutic intervention in gastrointestinal disorders. Front Pharmacol 12: 809350, 2021. doi:10.3389/fphar.2021.809350. 34992543 PMC8724579

